# Demonstration of a new entity of non-perforated appendicitis through studying cluster of appendicitis

**DOI:** 10.1038/s41598-022-16682-6

**Published:** 2022-07-21

**Authors:** Yitian Guo, Deqiang Ye, Guifang Yang, Guozhen Liu, Xiaochen Cui, Shiyun Tan, Yi Guo

**Affiliations:** 1 Department of Gastroenterology, Renmin Hospital of Wuhan University, Jiefang Road 238, Wuchang, Wuhan, 430060 Hubei China; 2 Department of Surgery, Infectious Disease Hospital Affiliated to Nanchang University (Nanchang Ninth Hospital), 167 Hongdu Middle Avenue, Qingshanhu District, Nanchang, 330002 Jiangxi China; 3 Department of Pathology, Zhongnan Hospital of Wuhan University, 169 Donghu Road, Wuchang, Wuhan, 430071 Hubei China; 4 Swedish Bellevue Primary Care Clinic, 1200 112th Ave, Bellevue, WA 98004 USA; 5 Department of Epidemiology and Biostatistics, Wuhan University School of Public Health, 115 Donghu Road, Wuchang, Wuhan, 430071 Hubei China; 6 Present Address: Department of Child Health Care, Wuhu Maternity and Child Health Care Hospital, Jiujiang, Wuhu, 241000 Anhi China

**Keywords:** Diseases, Gastroenterology, Medical research

## Abstract

Differential diagnosis and management for perforated appendicitis and non-perforated appendicitis are current hot topics. The aim of this study is to demonstrate a new entity of non-perforated appendicitis, “acute hemorrhagic appendicitis” through studying cluster of acute appendicitis among Tibetan students at a high school in central China. Over the 11-year period, there were 120 patients with more female patients (102 of 499, 20.4%) than male patients (18 of 474, 3.8%) among 973 Tibetan students. 117 patients’ clinical data were available. Clinical manifestations were identical to classic appendicitis. However, axilla temperature, white blood cell counts and neutrophil level were elevated mildly in 12 (10.3%), 19 (16.2%) and 12 (10.3%) patients respectively. Pathologically, the resected appendices exhibited focal or diffuse hemorrhages in mucosa and/or submucosa, and infiltration by eosinophil and by lymphocytes. No patients had perforated appendicitis. The median time from the onset to surgery was 3 days (IQR, 2–4). All patients were discharged with full recovery. In conclusion, “acute hemorrhagic appendicitis” represented a new entity of non-perforated appendicitis with unique cause and pathogenesis, which might be treated with antibiotics alone or self-limited. Studying the cluster is a reliable method to find new entity of appendicitis.

## Introduction

The pathological spectrum of the acutely inflamed appendix includes a wide range of noninfectious and infectious entities^1^. Etiology and pathogenesis of acute nonspecific appendicitis (the most common diagnosis made in this organ, which is often called acute appendicitis) remains uncertain^1,2^. It is still unsure how many different entities of acute appendicitis exist. Natural history of acute appendicitis has classically been described to often progress from an non-perforated appendicitis to perforated appendicitis^3^. Majority of the patients have elevated body temperature, total white blood cells counts and neutrophils in differential^4^. A new hypothesis has been proposed that perforated appendicitis and non-perforated appendicitis may be different entities with different natural history^5–11^. This has become modern classification^11^. Differential diagnosis and management for perforated appendicitis and non-perforated appendicitis are current hot topics^11–21^.

The classic description of acute appendicitis and the new hypothesis were derived from study of sporadic patients. The pathological examination of sporadic patients is targeted at a certain stage of different entities of acute appendicitis and the pathological changes of acute appendicitis cannot be seen continuously for the same patient. It is therefore that the classic description of natural history of acute appendicitis is deduced without direct evidence.

Acute appendicitis can be caused by different etiologies, therefore acute appendicitis should not be one entity, but a group of different entities. Cluster often results from common cause, therefore almost every patient in cluster belongs to the same entity and appeared in certain stage of same entity. Connecting each stage, we will know full natural histories of this entity and accordingly demonstrate whether or not perforated appendicitis and non-perforated appendicitis are different entities.

In 1984, a cluster of true appendicitis occurred in a town of the United States^22^. Four patients had perforated appendicitis in the 13 clustered patients. The cluster was paid a attention by the CDC and medical journals^23–25^. In 2004, we reported a more severe cluster of acute appendicitis at a high school in the city of Wuhan, central China^26^. As this kind of acute appendicitis had unique pathological features, namely, hemorrhage in the lamina propria and hyperplastic lymphoid follicles. We tentatively named this condition “acute hemorrhagic appendicitis”. No patient had perforated appendicitis in these patients. In 2012, Fusobacteria were also found in appendices of these patients^27^. To demonstrate a new disease, it is needed to determine whether or not the findings were simply happening by chance or truly characteristics of a new disease. If not reproduced, they may merely represent interesting observations^28^. In addition, It is often difficult to determine whether the reported patients are representative of all patients with the disease such that conclusions can be generalized^28^. In order to answer these questions, we have been looking for new cluster of acute appendicitis since beginning of 2005. We encountered a cluster of acute appendicitis among Tibetan students at a high school in the city of Nanchang, Jiangxi Province, eastern China.

The aim of this study was to demonstrate through studying cluster of acute appendicitis that “acute hemorrhagic appendicitis” was a new entity of non-perforated appendicitis occurring reproducibly in different provinces. A second aim was to confirm common settings of cluster of acute appendicitis and to provide suggestions to find new entity of acute appendicitis and identify the associated risk factors for conducting control and prevention of appendicitis in population.

## Materials and methods

### Study background and design

Since 1985, Tibetan students were enrolled at the schools of 20 provinces and municipalities of China. We learned that incidence rates of acute appendicitis increased at many of these schools. From beginning of 2005. We contacted four schools in four provinces where Tibetan students were enrolled and cluster of acute appendicitis occurred there for years. Finally, we selected Tibetan students as study population at a high school in the city of Nanchang as this school and the designated hospital for treating Tibetan students were willing to collaborate with this investigation.

We conducted observational study to investigate clinical, laboratory and pathological features of the patients among Tibetan students and also compared the features with those of the patients in the cluster of Wuhan to demonstrate that same entity of acute appendicitis can occur reproducibly in different province of China^26^. Our study was compliant with the CARE guidelines^29^.

We began this study in August of 2005. Retrospectively, we collected clinical data from January of 2000 to August of 2005. For patients occurring after August of 2005, we collected their clinical data after these patients were discharged from the hospital. Therefore, all clinical data after August of 2005 was also retrospective. Due to anonymity of clinical data and retrospective nature of this study, the informed consent from patients was waived by ethic board at Infectious Disease Hospital Affiliated to Nanchang University and Wuhan University School of Medicine. After preventive intervention to control appendicitis beginning in August of 2005, no further clusters of acute appendicitis occurred since 2011. We conducted surveillance from 2012 to July 2018^30^. During these periods, there were only 4 sporadic patients at this school. Therefore, we only summarized clinical, laboratory and pathological data of the clustered patients occurring from January of 2000 to end of 2010.

### Clinical, laboratory and pathological investigation

The study subjects included all patients with acute appendicitis from the high school during the period from January of 2000 to end of 2010. Case history, physical examination, laboratory tests, pathology examination were also recorded. 117 of the 120 patients operated on were from the designated hospital for Tibetan students, from which clinical and laboratory data were obtained for the study (The primary data were shown in supplementary Table S1 in which, “−” is negative result and “+”is positive result). Routine blood tests were performed before surgery for all patients. Patients were identified by reviewing hospital records. The study was approved by ethic board at Infectious Disease Hospital Affiliated to Nanchang University and Wuhan University School of Medicine in accordance with their guidelines and with the 1964 Helsinki declaration and its later amendments. Our study was registered in Chinese Clinical Trial Registry. Research Registry number is ChiCTR2100048111.

Patients was defined as those who had typical clinical manifestations of acute appendicitis and who had appendectomy and pathological features of acute appendicitis. All pathological slides from appendectomy were re-examined in Department of Pathology, Zhongnan Hospital of Wuhan University.

According to International Coding of disease (ICD-9), perforated appendicitis or appendiceal abscess or appendicitis with peritonitis were aggregated into a single category called “perforated appendicitis”^7,8^. Non-perforated appendicitis was defined as having any acute appendicitis except for perforated appendicitis or appendiceal abscess or appendicitis with peritonitis^7,8^.

On admission, the patient was diagnosed with acute appendicitis and was operated on immediately. Therefore, the time from onset to admission was combined with the time from admission to surgery into the time from onset to surgery. In most articles, the time from onset to surgery is represented by the mean. In order to facilitate comparison with other studies, our timeline is not only represented by median, but also by mean.

### Statistical analysis

Categorical variables were expressed as percentages and frequency rates. Continuous variables were expressed as mean, median, and interquartile range (IQR) values. Categorical variables were compared by the χ^2^ test or Fisher’s exact test was used when the data were limited. All statistical analyses were conducted using SPSS (Statistical Package for the Social Sciences) version 26.0 software (SPSS Inc). A 2-sided α of less than 0.05 was considered statistically significant.

## Results

### Epidemiological findings

The high school is located in an urban district of the City of Nanchang, Jiangxi province. From 1985, the school enrolled Tibetan students. Length of schooling was 4 years. From 1998, the incidence rate began to increase among Tibetan students. Since 2000, the patients had been admitted to the designated hospital. From January of 2000 to end of 2010, 973 Tibetan students were enrolled at this school during this period. Among them, 120 (12.3%) students suffered from acute appendicitis. There was a significant difference (χ^2^ = 62.28, *P* = 0.001) between incidence rates in male (3.8%, 18 of 474) and female (20.8%, 102 of 499). Patients’ age ranged from 12 to 17 years. The mean age (SD) was 14.3 (1.1) years. The patients occurred in cluster. Many of them had a history of mutual contact before the onset of the disease**.**

### Clinical, laboratory and pathological findings in clustered patients

117 of the 120 patients operated on were from the designated hospital, from which clinical and laboratory data were obtained for the study; the other 3 patients, who were operated on but whose clinical and laboratory data were not obtained, were not hospitalized at the designated hospital. The clinical and laboratory features of patients among Tibetan students and comparison with Wuhan clustered patients were presented in Table 1. The patients’ clinical symptoms and signs had features of classic acute appendicitis. The most common manifestations were right lower quadrant pain or midabdominal pain migrating to the right lower quadrant (117[100%]), tenderness at or near McBurney’s point (117[100%]), rebound tenderness (58[49.6%]) and involuntary muscle spasm (60[51.3%]). Less manifestations were nausea (34[29.1%]) and vomiting (14[12.0%]). However, the patients’ results of body temperature and routine blood tests exhibited an obvious different pattern, namely, of 117 patients, only 12 (10.3%) had axilla temperature more than 37 °C. White blood cell counts and neutrophil percentage were elevated mildly in only 19 (16.2%) patients and 7 (6.0%) patients respectively. (Note for Table 1: ^1^The patients’ fever range: 37.1 °C to 38.9 °C. Only two patients had axilla temperature more than 38 °C. ^2^For white blood cell counts, reference value:4 × 10^9^/L to 10 × 10^9^/L. The patients’ increased white blood cell count range: 10.5 × 10^9^/L to 15 × 10^9^/L. ^3^For percentage of neutrophil, according to report of blood routine examination, reference value:40% to 80%. Increased percentage of neutrophil range: 84% to 85%. ^4^“−” in Table 2 means that the associated data were not shown in reference^26^).

**Table 1:** Clinical and laboratory features of Tibetan and Wuhan clustered patients.

Characteristics	Wuhan patients (n = 29)	Nanchang patients (n = 117)	*P* value
**Gender**			
Male	7 (24.1)	17 (14.5)	0.210
Female	22 (75.9)	100 (85.5)	
**Signs and symptoms**			
Fever	4 (13.8)	12 (10.3)	0.830
Nausea	–	34 (29.1)	–
Vomiting	–	14 (12.0)	–
Right lower quadrant pain or midabdominal Pain migrating to the right lower quadrant	29 (100.0)	117 (100.0)	–
Tenderness in at or near McBurney’s point	29 (100.0)	117 (100.0)	–
Rebound tenderness	20 (69.0)	58 (49.6)	0.060
Involuntary muscle spasm	5 (17.2)	60 (51.3)	0.001
Diarrhea	3 (10.3)	0 (0.0)	0.007
**Laboratory test**			
White blood cell counts increase	6 (20.7)	19 (16.2)	0.570
Neutrophil percentage	12 (41.4)	7 (6.0)	< 0.001

Categorical variables were compared by the χ^2^ test or Fisher’s exact test was used when the data were limited. A 2-sided α of less than .05 was considered statistically significant.

**Table 2:** Pathological features of 116 Tibetan patients and comparison with Wuhan clustered patients.

Characteristics	Wuhan patients (n = 29)	Nanchang patients (n = 116)	*P* value
Hemorrhage in the lumen of the appendix	20 (70.0)	77 (66.4)	0.79
Diffuse or focal hemorrhage in the lamina propria or hyperplastic lymphoid follicles	23 (79.3)	77 (66.4)	0.18
Eosinophilic Infiltration in lamina propria, submucosa, lymphoid follicles and muscle layers	14 (48.3)	78 (67.2)	0.06
Lymphocytic infiltration in epithelium and crypt	–	67 (57.8)	–
Lymphocytic infiltration in serosa layer and subserosa	5 (17.2)	50 (43.1)	0.01
Fecalith	–	46 (39.7)	–
Parasite eggs in appendices	0 (0.0)	0 (0.0)	–

Categorical variables were compared by the χ^2^ test or Fisher’s exact test was used when the data were limited. A 2-sided α of less than .05 was considered statistically significant.

The clinical and laboratory features between Tibetan clustered patients and Wuhan clustered patients were not significantly different except involuntary muscle spasm and neutrophil percentage. The reason why neutrophil percentage between the Tibetan clustered patients and the Wuhan clustered patients differed significantly may be that their reference value was different. For percentage of neutrophil of Tibetan clustered patients, reference value: 40% to 80% according to report of blood routine examination of the designated hospital, Table 2. For Wuhan clustered patients, reference value: 50% to 70%.

During surgery, hyperemia and edema were observed on the appendix of each patient. However, no perforation was found and no purulent surface exudates were identified except for 7 patients.

**Figure 1:**
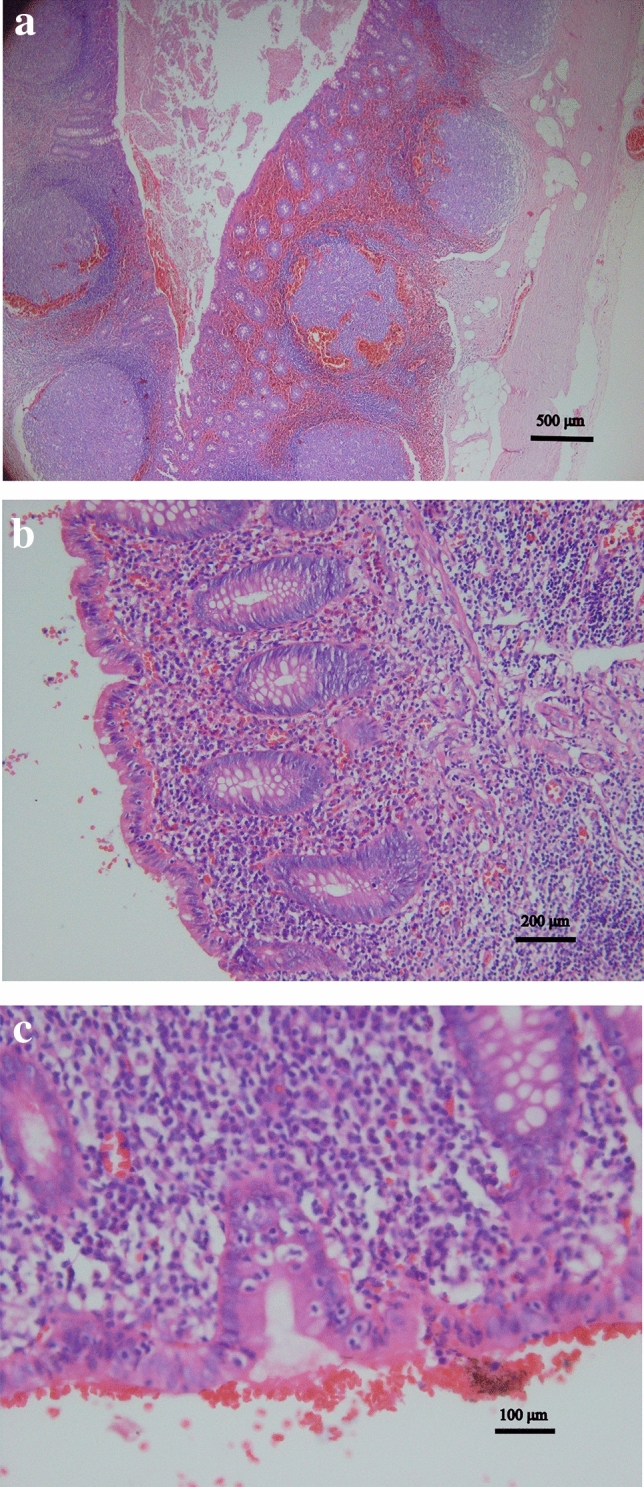
Pathological features of acute appendicitis in Tibetan students, as shown by hematoxylin and eosin stain. (**a**) diffuse hemorrhages in the lamina propria and lymphoid follicles as well as in the lumen of the appendix . (**b**) Infiltration of the lamina propria by scattered eosinophils. (**c**) The epithelium is infiltrated by lymphocytes.

116 patients were exanimated pathologically. The pathological findings of these patients and comparison with Wuhan clustered patients were presented in Table 2. (Note for Table 2. The primary data were shown in supplementary Table S1. “−” in Table 2 means that the associated data were not shown in reference^26^). The most common findings of the patients among Tibetan students were hemorrhage in the lumen of the appendix (77[66.4%]), diffuse or focal hemorrhage in the lamina propria or hyperplastic lymphoid follicles (77[66.4%]). Eosinophilic Infiltration in lamina propria, submucosa, lymphoid follicles and muscle layers (78[67.2%]). Lymphocytic infiltration in epithelium and crypt (67[57.4%]). 46 (39.7%) patients had fecalith. Parasite eggs were not found. The pathological features were shown in Fig. 1.

The pathological features between Tibetan clustered patients and Wuhan clustered patients were not significantly different except lymphocytic infiltration in serosa layer and subserosa, which is not main features in pathology.

The pathological diagnosis for Tibetan clustered patients and comparison with Wuhan clustered patients were shown in Table 3. Except for 6.9% (8 of 116) patients had acute suppurative appendicitis and 1.7% (2 of 116) had acute gangrenous appendicitis, 89.7% (104 of 116) Tibetan clustered patients had “acute hemorrhagic appendicitis”. No perforated appendicitis occurred. Among Wuhan clustered patients, 6.9% (2 of 29) patients had acute suppurative appendicitis and 93.1% (27 of 29) patients had “acute hemorrhagic appendicitis”. There was no significant difference in acute suppurative appendicitis and “acute hemorrhagic appendicitis” between Tibetan and Wuhan clustered patients.

**Table 3:** The pathological diagnosis for Tibetan clustered patients and Wuhan clustered patients.

Diagnosis	Wuhan patients (n = 29)	Tibetan patients (n = 116)	*P* value
“Acute hemorrhagic appendicitis”	27 (93.1)	104 (89.7)	0.74
Acute suppurative appendicitis	2 (6.9)	8 (6.9)	1.00
Acute gangrenous appendicitis	0 (0.0)	2 (1.7)	1.00

Categorical variables were compared by the χ^2^ test or Fisher’s exact test was used when the data were limited. A 2-sided α of less than .05 was considered statistically significant.

**Figure 2:**
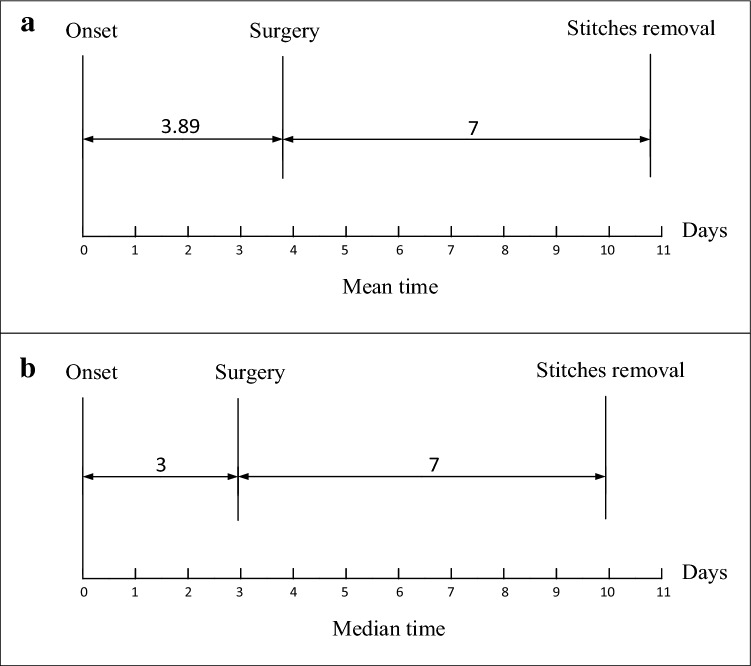
Timelines of “acute hemorrhagic appendicitis” after onset of illness.

The timeline was shown in Fig. 2. The median time from the onset to surgery was 3 days (IQR, 2–4) and mean time was 3.89 days (± 4.33) respectively. Antibiotics were routinely administered after surgery. All patients had their stitches removed 7 days after surgery and they were discharged with full recovery.

## Discussion

We presented the clinical, laboratory and pathological features of the patients among Tibetan students. It is generally recognized that acute appendicitis is a sporadic, noninfectious disease. pathological examination usually exhibits purulent inflammation with neutrophils. The majority of the patients have elevated white blood cell counts and/or neutrophil percentage whereas the patients among Tibetan students exhibited different features quite obviously: (1) Gross pathological examination of appendices exhibited only minimal purulent change and microscopic examination exhibited hemorrhage with infiltration by eosinophils and lymphocytes. The features can be distinguished from that of the known infectious and noninfectious acute appendicitis and the clustered patients in the United States^1,22^. (2) In clinical data, only a minority of the patients had mildly elevated body temperature and/or white blood cell counts and/or neutrophil percentage. (3) In epidemiology, there were the occurrence of clustering. Incidence of female patients was more common than male patients and many of them had a history of mutual contact before the onset of acute appendicitis. The above features were identical to the clustered patients with “acute hemorrhagic appendicitis” in Wuhan. According to these similarities, it can be considered that acute appendicitis among Tibetan clustered patients and Wuhan clustered patients belongs to the same entity of acute appendicitis. It demonstrated that “acute hemorrhagic appendicitis” did not happen by chance, but a new entity of acute appendicitis which can occur reproducibly in different provinces. As we analyzed all Tibetan clustered patients and Wuhan clustered patients, so the patients’ features were representative for “acute hemorrhagic appendicitis”. Natural history of Tibetan clustered patients and Wuhan clustered patients did not progress from a non-perforated appendicitis to perforated appendicitis, therefore “acute hemorrhagic appendicitis” is an independent non-perforated entity from perforated appendicitis.

The first English report of “acute hemorrhagic appendicitis” appeared in 1964^31^. 252 sporadic patients with this disease occurred in Beckley and Oak hill hospitals, Beckley, West Virginia during a ten year period from 1951 to 1961. The patients mostly were children and adolescents, with more males than females. Of them, 76 represented pure hemorrhagic appendicitis and 186 had purulent-hemorrhagic appendicitis. There was no record of eosinophil infiltration pathologically. Majority of patients had elevated body temperature and white blood cell counts. The above clinical and pathological features explained that “acute hemorrhagic appendicitis” in Tibetan clustered patients and Wuhan clustered patients was different from the sporadic “acute hemorrhagic appendicitis”.

The pathogenesis of acute appendicitis has not been completely understood. Obstruction of appendiceal lumen is believed to be the main cause^1,2,4^, which may be caused by fecalith or parasites or lymphoid hyperplasia and so forth. This causes an increase in pressure, engorgement and stasis leading to necrosis and eventually perforation. Pathologically, resected appendices exhibited purulent information with neutrophils. However, pathological features of “acute hemorrhagic appendicitis” were hemorrhage with infiltration by eosinophils and lymphocytes although 39.66% (46 of 116) Tibetan clustered patients had fecalith. The absence of parasite eggs in patients’ appendices explain that appearance of eosinophil was not associated with parasitic infection. The above features suggested that the cause and pathogenesis of “acute hemorrhagic appendicitis”may be different from classic acute appendicitis.

There was no record of stool hemorrhage in the patients’ charts. Platelet count, prothrombin time, activated partial thromboplastin time, throbmin time and fibrinogen level were normal in all patients. Physical examination did not find hemorrhagic symptoms and signs. These results suggest that “acute hemorrhagic appendicitis” is neither a local manifestation of general hemorrhagic diseases nor a manifestation of other intestinal hemorrhagic diseases.

It is generally believed that risk of perforation increases as time elapses from onset of appendicitis to treatment, which cause increased patient mortality. Some studies reported respectively that the duration from the onset of appendicitis to admission of 12, 24 and 36 h increased risk for perforation respectively^32–35^. Tibetan clustered patients were operated on immediately after diagnosis as appendicitis on admission, therefore the time from onset to surgery was very close to the time from onset to admission. Although their median time from the onset to surgery was 3 days (IQR, 2–4) and mean time was 3.89 days (± 4.33) respectively, none of patients had perforation. It showed that delayed appendectomy may be safe for patients with “acute hemorrhagic appendicitis”.

In recent years, a clinical classification is used to stratify management for acute appendicitis based on simple (non-perforated appendicitis) and complex (gangrenous and perforated) inflammation^11^. Some patients with simple appendicitis might be self-limited or respond to antibiotics alone^11^. Except 2 patients with acute gangrenous appendicitis, all of Tibetan clustered patients were simple appendicitis, suggesting that the treatment of “acute hemorrhagic appendicitis” might be administered with antibiotics alone. Among Wuhan clustered patients, 10 patients recovered without surgery^26^, suggesting that “acute hemorrhagic appendicitis” might also be self-limited. Since fecalith may be the cause of appendicitis, therefore non-surgical treatment or self-limitation would be more advisable for non-fecalith “acute hemorrhagic appendicitis”.

How to formulate diagnosis of “acute hemorrhagic appendicitis” in patients who are not treated surgically is a challenging issue, which is related to the probability of predicting the success of non-surgical treatment. We assume that there are two approaches to solve this issue in future.

1. Founding out the proportion of “acute hemorrhagic appendicitis” in all patients with normal body temperature, white blood cell counts, neutrophil level and longer time from onset to admission. After finding out the proportion of “acute hemorrhagic appendicitis”, we can calculate the probability of “acute hemorrhagic appendicitis” when patients on admission have normal body temperature, white blood cell counts and so forth.

In addition, we can also use ultrasound, computed tomography and magnetic resonance to examine features of appendix of “acute hemorrhagic appendicitis” and compare these features with other kind of appendicitis. If “acute hemorrhagic appendicitis” has different features from other kind of appendicitis, these features can be used for diagnosis of “acute hemorrhagic appendicitis”.

2. Finding out the precipitating infectious agent of “acute hemorrhagic appendicitis” and making specific diagnosis. Clusters are often caused by common factors and are often associated with infectious agents. Regarding clustering of acute appendicitis, the Centers for Disease Control and Prevention (CDC) published editorial note that the cluster offered a unique opportunity to identify possible risk factors and to search for precipitating infectious agents, and encouraged reporting such cluster to CDC^23^. Since appendicitis is an intestinal disease, the potential infectious agent may be isolated from stool or removed appendix of the patients with clinical features of “acute hemorrhagic appendicitis”, preferably from new clustered patients with “acute hemorrhagic appendicitis”. If successful, it can be used for early diagnosis, such as detecting the patient's stool to find specific pathogens and serum sample to find specific antibodies and so on.

According to literature index, clusters of acute appendicitis also occurred in five other provinces and autonomous regions of China^36–42^, which often occurred in collective living units, such as schools and military camps, it is feasible that focusing on collective living units and finding new cluster of acute appendicitis to demonstrate new entity. Because acute appendicitis is not endemic disease, cluster of acute appendicitis can also occur widely worldwide. This suggests that the discovery of new entities of acute appendicitis through studying cluster of acute appendicitis can be generalized.

Strengths: (1) We obtained full natural history of “acute hemorrhagic appendicitis”through studying cluster of acute appendicitis. Intra-cluster consistency existed, namely, the vast majority of patients had similar features in Tibetan and Wuhan clustered patients. Inter-cluster consistency also existed, namely, the vast majority of patients had similar features between Tibetan and Wuhan clustered patients. “acute hemorrhagic appendicitis” can occur reproducibly in different provinces, therefore the conclusion that “acute hemorrhagic appendicitis” is a new entity of non-perforated appendicitis should be reliable.

Limitation: (1) As the pathogen was not isolated, we cannot confirm that “acute hemorrhagic appendicitis”was caused by infectious agents. (2) For patients with “acute hemorrhagic appendicitis”, we have not find a method of early diagnosis before surgery.

Conclusion: We examined the clinical, laboratory, and pathological features of a group of patients with “acute hemorrhagic appendicitis” among Tibetan students from a high school in eastern China. All features suggest that this cluster of acute appendicitis represents a new entity of non-perforated appendicitis, with milder clinical and laboratory features and unique pathological findings, namely, hemorrhage with infiltration by eosinophil and lymphocytes. The above features were identical to the clustered patients with “acute hemorrhagic appendicitis” in Wuhan, central China. It demonstrated that “acute hemorrhagic appendicitis” did not happen by chance, but truly characteristics of a new kind of acute appendicitis. The cause and pathogenesis of “acute hemorrhagic appendicitis” may be different from classic acute appendicitis, which might be treated with antibiotics alone or self-limited. Studying the cluster of acute appendicitis can help to discover new entities of acute appendicitis and associated pathogens. Future research should focus on collective living units and finding new cluster of acute appendicitis to demonstrate new entity, identify cause of the disease and pathogenesis for conducting control and prevention of appendicitis in population.

## Supplementary Information

Supplementary Information.

## Data Availability

Our data was submitted with our manuscript in Supplementary Table S1 for clinical features. If our manuscript can be published, we suggested that the data is open to reader.
